# Poster Session I - A127DEVELOPMENT OF INTERACTIVE WEB-BASED ADVANCED MEDICATION THERAPY TOOLS FOR USE BY CANADIAN INFLAMMATORY BOWEL DISEASE NURSES

**DOI:** 10.1093/jcag/gwaf042.127

**Published:** 2026-02-13

**Authors:** C Johnston, J Stretton, M Martin, N Rohatinsky

**Affiliations:** Gastroenterology, Foothills Medical Centre, Calgary, AB, Canada; Gastroenterology, St Joseph’s Healthcare Hamilton, Hamilton, ON, Canada; Gastroenterology, Foothills Medical Centre, Calgary, AB, Canada; Nursing, University of Saskatchewan, Saskatoon, SK, Canada

## Abstract

**Background:**

Crohn’s disease and ulcerative colitis, collectively termed inflammatory bowel disease (IBD), are chronic lifelong autoimmune conditions that are on the rise in Canada. Overall, the prevalence of IBD in 2023 is estimated to be 825 per 100,000 (410 per 100,000 for Crohn’s disease, and 414 per 100,000 for ulcerative colitis and IBD-u). Management of IBD often involves sustained medication therapy to manage inflammation and promote mucosal healing. Numerous advanced medication therapies are available for use and many others continue to be introduced. These advanced therapies can have different pre-administration testing requirements, delivery modalities, precautions, and adverse effects. Selecting the most appropriate advanced medication therapy can be overwhelming for persons with IBD and shared decision making between the patient and IBD provider can be enhanced by using a web-based tool that outlines key pieces of information.

**Aims:**

To provide health professionals with a comprehensive tool to promote decision making for advanced therapies in IBD.

**Methods:**

Recognizing a gap in information to facilitate advanced medication therapy decision making for both patients and providers, members of the Canadian IBD nurses (CANIBD) organization developed a web-based tool (https://canibduc.ca/). Two senior IBD Nurse Practitioners worked over three months reviewing product monographs (and consensus statements) to organize this information in easily accessible format(tools). These tools review the medication class and action, method of administration and location for administration, testing required before and during therapy, side effects in addition to considerations for special populations (pediatrics/elderly/breastfeeding/pregnancy). The web-based tools are accessible to IBD providers across Canada.

**Results:**

Informal feedback was obtained by CANIBD board members from approximately 25 IBD nurses. Based on preliminary feedback, the tools provide a quick and comprehensive overview of medication treatment options that can be used by IBD nurses and other health professionals. The tools are user-friendly and presented in a manner that are easy to review with persons with IBD to promote shared and informed decision making. The tools will be updated regularly as new treatment options become available. More formal evaluation is planned to determine the tools’ effectiveness and usability from both patient and provider perspectives.

**Conclusions:**

These web-based tools allow providers access to comprehensive IBD advanced medication therapy materials to promote information sharing with persons living with IBD so that informed, shared decision making can occur. These materials also promote CANIBD’s goals for patient advocacy and collaboration.

**Funding Agencies:**

None

